# Generic and Flexible Unmanned Sailboat for Innovative Education and World Robotic Sailing Championship

**DOI:** 10.3389/frobt.2021.630081

**Published:** 2021-03-11

**Authors:** Shaolong Yang, Chuan Liu, Ya Liu, Jinxin An, Xianbo Xiang

**Affiliations:** ^1^ School of Naval Architecture and Ocean Engineering, Huazhong University of Science and Technology, Wuhan, China; ^2^ Hubei Key Laboratory of Naval Architecture and Ocean Engineering Hydrodynamics, School of Naval Architecture and Ocean Engineering, Huazhong University of Science and Technology, Wuhan, China

**Keywords:** world robotic sailing championship, unmanned sailboat, pixhawk, open-source design, innovative education

## Abstract

Over the past two decades, scholars developed various unmanned sailboat platforms, but most of them have specialized designs and controllers. Whereas these robotic sailboats have good performance with open-source designs, it is actually hard for interested researchers or fans to follow and make their own sailboats with these open-source designs. Thus, in this paper, a generic and flexible unmanned sailboat platform with easy access to the hardware and software architectures is designed and tested. The commonly used 1-m class RC racing sailboat was employed to install Pixhawk V2.4.8, Arduino Mega 2,560, GPS module M8N, custom-designed wind direction sensor, and wireless 433 Mhz telegram. The widely used open-source hardware modules were selected to keep reliable and low-cost hardware setup to emphasize the generality and feasibility of the unmanned sailboat platform. In software architecture, the Pixhawk V2.4.8 provided reliable states’ feedback. The Arduino Mega 2,560 received estimated states from Pixhawk V2.4.8 and the wind vane sensor, and then controlled servo actuators of rudder and sail using simplified algorithms. Due to the complexity of introducing robot operating system and its packages, we designed a generic but real-time software architecture just using Arduino Mega 2,560. A suitable line-of-sight guidance strategy and PID-based controllers were used to let the autonomous sailboat sail at user-defined waypoints. Field tests validated the sailing performance in facing WRSC challenges. Results of fleet race, station keeping, and area scanning proved that our design and algorithms could control the 1-m class RC sailboat with acceptable accuracy. The proposed design and algorithms contributed to developing educational, low-cost, micro class autonomous sailboats with accessible, generic, and flexible hardware and software. Besides, our sailboat platform also facilitates readers to develop similar sailboats with more focus on their missions.

## Introduction

Ocean is vast. Until now, humans just have only explored a limited ocean area in view of 70% of the earth's surface. Over the past decades, the invention and implementation of marine robots contribute to pushing ocean data science into a new area. Versatile marine robots speed up ocean exploration in both surface and underwater environments. Among various marine robots, autonomous sailboats attract great attention due to the possible infinite endurance (Silva et al., 2019). Up to now, limited to the available energy forms for mobile robots, most marine robots are propelled using electric motors or diesel engines, which are dependent on the onboard capacity of battery or fuel tank. Thus, for the long term and large area ocean observation in remote sea areas, sailboats would be a perfect choice for ocean data observation.

Motivated by this laudable goal, several autonomous sailboats were developed over the past two decades. An early try in robotic sailboats was Atlantis with wing-sail, which was designed by Elkaim and Kelbley in 2001 (Elkaim and Kelbley, 2006). This catamaran sailboat demonstrated the autonomous control capability of segmented trajectory following. This technology was then used to develop another winged catamaran called HWT-X1, which was supported by harbor wing technologies company (Hugh Elkaim and Lee Boyce, 2007). After that, in 2014, Ocean Aero company designed a mono-hull wing sailboat called Submaran (Mayerfeld, 2017), which is capable of both surface and subsurface travel. Meanwhile, another company Saildrone also developed a similar wing sailboat, which was already used to collect ocean data for US national oceanic and atmospheric administration (Voosen, 2018; Gentemann et al., 2020).

**FIGURE 1:**
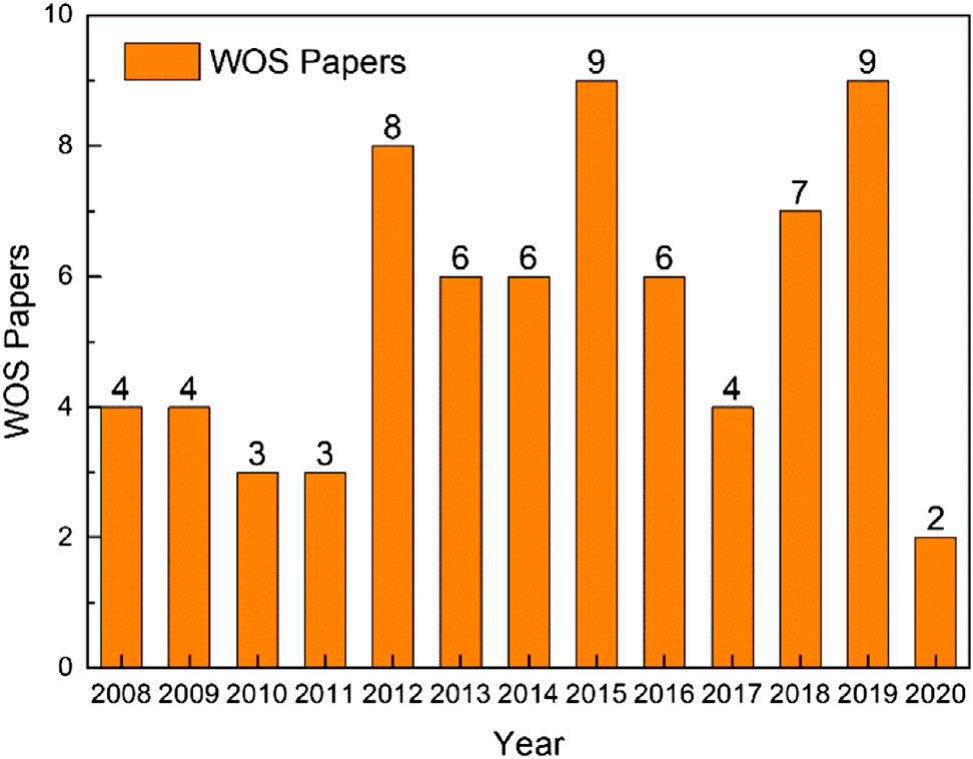
A bibliometric investigation in the topic of autonomous sailboat research.

Although autonomous sailboats are booming in the industrial field, the research about robotic sailboats seems to lag behind in academia. A bibliometric investigation was made using the web of science (WOS) core collection database, with an advanced search equation of TS =(“autonomous sailboat” or “unmanned sailboat” or “robotic sailboat” or “autonomous sailing” or “robotic sailing”) and timespan from 1985 to 2020. The results are shown in Figure 1. Up to now, a total of 71 papers are found related to the topic, and the authors from France, USA, and Portugal contributed 43 papers accounting for 60%. Therefore, global researchers did not widely engage in autonomous sailboat research. Nevertheless, each year, a lot of international sailboat activities were held worldwide, like Olympic sailing sports, world radio sailing racings, and America's Cup sailing yachts competitions. These activities really attract lots of sailboat players, engineers, and fans to dedicate to improving sailing sports and technologies. So, how to engage these people’s attention in promoting the development of autonomous sailboat technologies becomes a question worth thinking about.

Aiming at stimulating the development of autonomous wind propelled marine robots, the World Robotic Sailing Championship (WRSC) and the International Robotic Sailing Conference (IRSC) were held since 2008. The WRSC is an international competition open to fully autonomous and unmanned sailing boats up to 4 m in length. Like the WRSC competition, there are also two other autonomous sailboat competitions, including SailBot and Microtransat. Each year, lots of sailboat research teams around the world get together in this competition, and discuss scientific and engineering issues related to the design and control of robotic sailboats. For example, the last 12th WRSC competition (2019) was held in Ningbo, China. Our team participated in this activity with two sister autonomous sailboats named SAILAMRS I and II. Competed with more than 20 international teams, the SAILAMRS I and II finally won the second prize.

**FIGURE 2:**
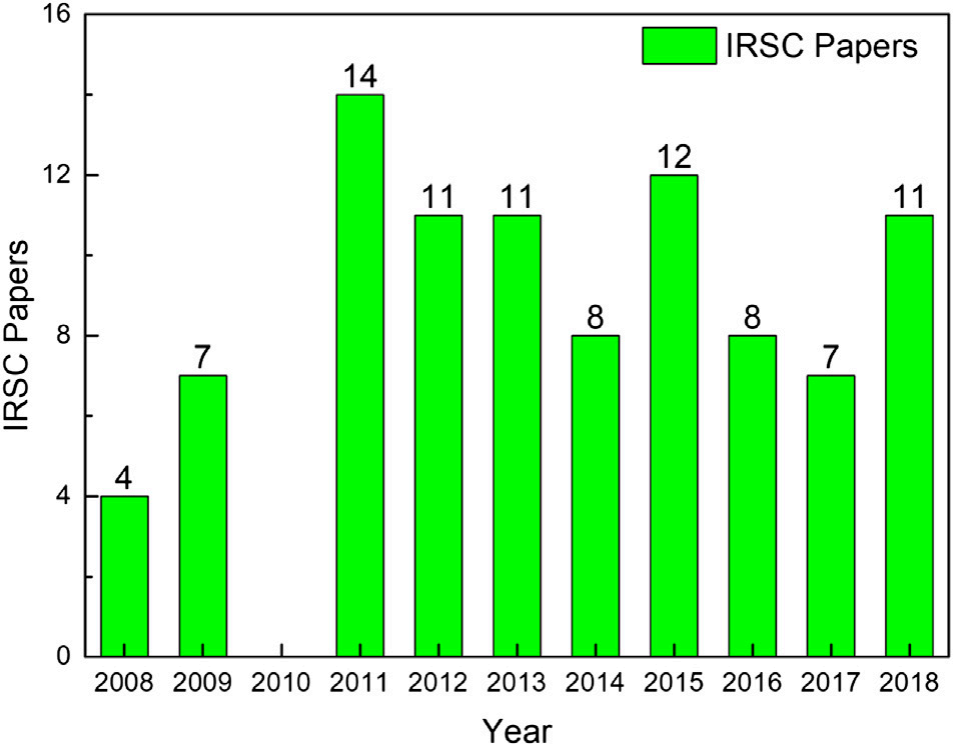
Summary of papers published in IRSC activities.

Driven by the annual WRSC and the accompanying IRSC, autonomous sailboat research received more widespread attention. A statistic result of papers published in annual IRSC is shown in Figure 2. It can be seen that more specialized papers focused on autonomous sailboats were published in IRSC annually, compared with WOS papers. A total of 93 papers were published in last ten IRSC activities, which included topics of sailboat platforms and applications, mission planning, obstacle avoidance, and controllers and sensors.

Among these papers, several autonomous sailboats were well introduced and had done great work in WRSC competitions (Stelzer and Jafarmadar, 2011; Yu et al., 2018; Silva et al., 2019), including ASV Roboat, Avalon, FASt, IBoat, A-Tirma, Arrtoo, Vaimos, Aeolus. As shown in Table 1, the comparisons of some typical WRSC autonomous sailboats with our SAILAMRS was made. Prior autonomous sailboats generally had a large dimension with rich hardware modules, which were more suitable for performing novel scientific research. However, although several WRSC autonomous sailboats disclosed their design schemes and source codes online, it is actually hard for interested researchers or learners to make a follow-up sailboat. The main barriers may include but not limited to custom-designed boat forms, complicated hardware and software architectures, complex controlling strategies, as well as high investment activities. Therefore, because of these barriers, the present paper presents a generic and flexible unmanned sailboat design. The basic idea is to provide a hardware and software scheme for interested researchers to build their own autonomous sailboat. Based on this worthwhile goal, a 1-m class autonomous sailboat was designed with the commonly-used remote control (RC) sailboats, which could participate in WRSC activities.

**TABLE 1:** Comparison of WRSC autonomous sailboats.

Name	Dimension	Hardware	Algorithm	Reference
ASV roboat	LOA: 2 m	Rabbit 3,000 microprocessor, MicroMag 3-axis compass, trimble IQ GPS, AC-12 DGPS, accelerometer	Fuzzy logic controller	Klinck et al. (2009)
	Beam: 0.36 m			
	Draft: 1.5 m			
	Mass: 300 kg			
Avalon	Length:3.95 m	Computer (MPC21, 500 MHz), GPS, IMU, wind sensor, AIS, INMARSAT satellite network	Grid-based A* path planning algorithm, finite-state machine control of motion	Giger et al. (2009)
	Beam: 1.40 m			
	Draft: 0.25 m			
Arrtoo	Length: 2 m	Main microcontroller (GPS, compass, wind sensor) and comms microcontroller (iridium, ZigBee, satellite tracker)	—	Miller et al. (2013)
	Beam:0.479 m			
	Draft: 0.83 m			
Aeolus	Length: 1 m	Autopilot controller (PIXHAWK) and weather station (AIRMAR WS-200WX)	a custom designed nonlinear controller rule-based sail controller, and a LQR tack controller	Wirz et al. (2015)
SAILAMRS	Length: 1 m	Arduino mega 2,560, pixhawk V2.4.8, wireless radio module (433 Mhz), radio receiver (2.4 GHz), GPS module (M8N), wind vane sensor	Line of sight guidance, and PID rudder and sail controlers	In this study
	Beam: 0.17 m			
	Draft: 0.42 m			
	Mass: 4.4 kg			

The remainder of the article is organized as follows. *One-Meter Class Autonomous Sailboat Setup* introduced the low-cost open-source hardware setup, which can be easily accessed by most robotics labs. *Autonomous Sailboat Software Design* presents the modular software structure, realizing the path planning and path following for autonomous sailboats. Beginners only need to focus on the simple Arduino controlling programming, while the open-source Pixhawk firmware could solve the stable state estimation and communication. *Autonomous Sailboat Sailing and Controlling* details the basic sailing principle of sailboats, as well as the simplified controllers for rudder and sail. Based on the sailboat characteristics, the guidance and control algorithms for the autonomous sailboat are proposed. *World Robotic Sailing Championship Mission-Oriented Verification in the Lake Test* discusses the field test results of the proposed autonomous sailboat. The typical WRSC mission tasks under a realistic environment were checked using the autonomous sailboat, which showed acceptable performance in view of tracking trajectories. Finally, *Conclusion* summarizes the article, and indicates the proposed generic sailboat platform in this paper would be a perfect platform for inspiring more researchers and students to be interested in robotic sailboats.

## One-Meter Class Autonomous Sailboat Setup

**FIGURE 3:**
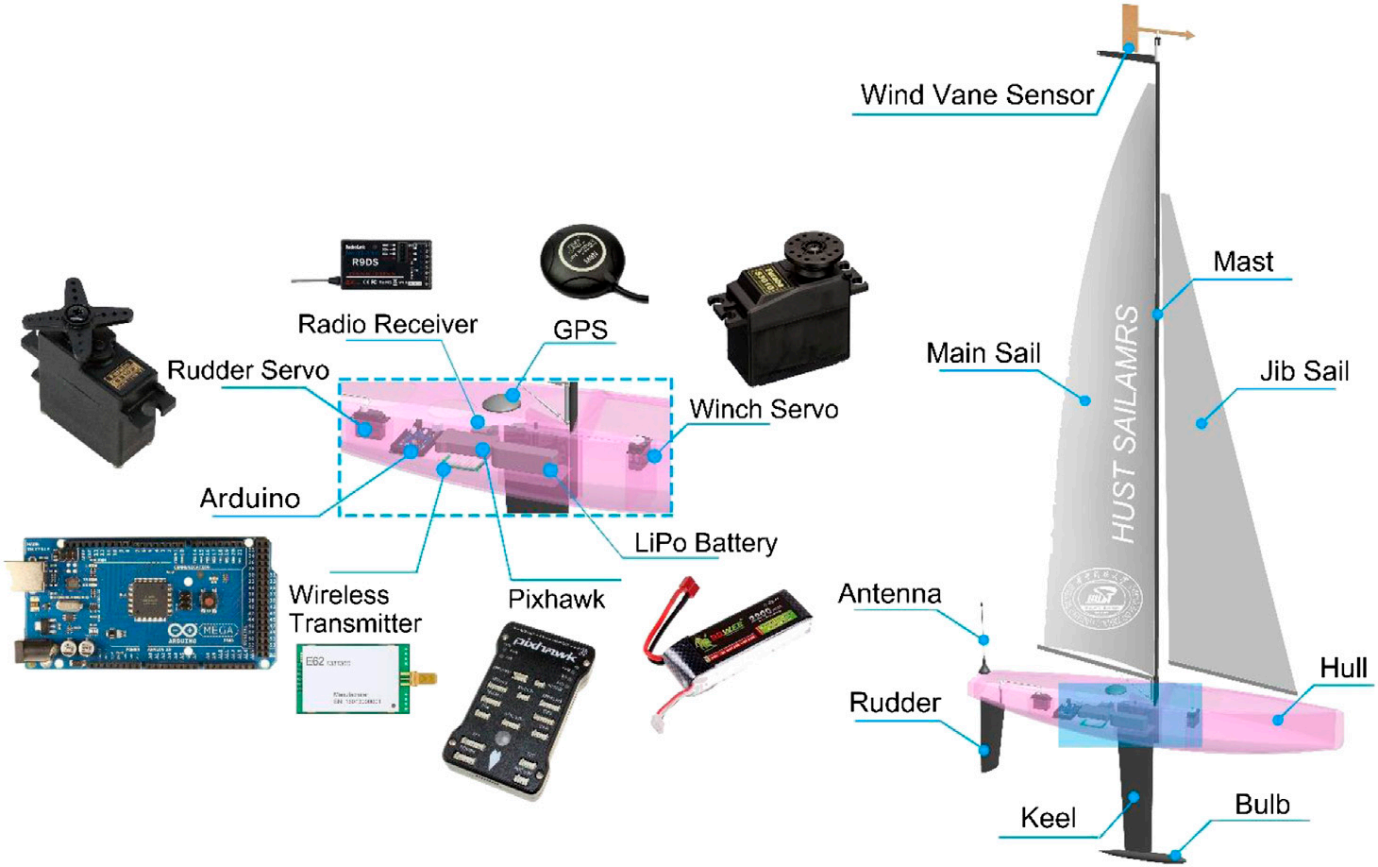
Components layout diagram.

A generic and flexible autonomous sailboat platform is the most important foundation for wide engagement and education in autonomy research of unmanned sailboat. To this end, we proposed a 1-m class autonomous sailboat setup with various reliable and easy-access hardware modules, as shown in Figure 3. The 1-m class autonomous sailboat designed by our team is called SAILAMRS. Components of the 1-m class autonomous sailboat could be divided into boat, sail, and electronics. The boat include hull, rudder, keel and bulb. The sail contain the mast, main sail, jib sail, and rigs. The basic boat and sail components are just the similar to the commonly-used 1-m class RC sailboat, so that more interested people could have easy access to the RC sailboat hardware. Compared with commercially available RC sailboats, the electronics in the 1-m class autonomous sailboat are the most unique part. They are mainly Arduino Mega 2,560 microcontroller, Pixhawk V2.4.8 autopilot, wireless radio module (E62-433T30D, 433 Mhz), radio receiver (R9DS, 2.4 GHz), GPS integrated with compass module (M8N), wind vane sensor, winch servo (Futaba S3010), rudder servo (Futaba S3102), and Lipo battery. All these electronic components are installed in the hull with a waterproof structure. Additionally, a 3D printed supporting structure was employed to fasten various components and connectors.

### Bill of Materials

Due to the overall budget and transportation convenience, the educational sailboat of SAILAMRS is limited to a length of 1 m. The basic dimensions and capacities are displayed in Table 2. It can be easily transported via public transport. Furthermore, mission-oriented payloads could be installed on the sailboat, including conductivity, temperature, depth (CTD) sensors, and portable weather stations. A detailed bill of materials (BOM) is listed in Table 3, in which the overall cost is less than ¥10,000RMB. From the BOM spreadsheet, it can find that most of the sailboat parts were low cost, and could be easily assessed by mobile robotics research labs. The open-source hardware (e.g., Arduino Mega 2,560 and Pixhawk V2.4.8) contributes to the low budget, due to the wide applications in universities and companies all over the world. For the sailboat, a 1-m class RC racing sailboat was used and modified to be an autonomous one, so that more fans of RC racing sailboat could have a chance to participate in the WRSC activity.

**TABLE 2:** Basic information of SAILAMRS sailboat.

Name	Value	Name	Value
Length	1 m	Draft	0.42 m
Width	0.17 m	Sail area	0.634 m^2^
Weight	4.5 kg	Top speed	5 kn
Battery	11.1 V 2200 mAh	Payload capacity	1 kg

**TABLE 3:** Bill of materials of SAILAMRS sailboat.

Component	Description	Cost per unit currency	Sources of Materials
Arduino mega 2,560	Microcontroller (16 MHz clock, 8 KB SRAM)	¥200RMB	Semiconductor
Pixhawk V2.4.8	State observer (STM32F427 and STM32F103)	¥400RMB	Semiconductor
GPS u-blox M8N	Gnss receiver	¥200RMB	Semiconductor
Battery	Lipo pack 2200 mAh 3S25C	¥80RMB	Battery
Wireless module	E62-433T30D (433 MHz)	¥200RMB	Semiconductor
Winch servo	Futaba S3010	¥200RMB	Motor
Rudder servo	Futaba S3102	¥200RMB	Motor
Wind vane sensor	Magnetic rotary encoder AS5040	¥200RMB	Other
Sailboat	Hull, keel, rudder, sail and rigging	¥8000RMB	Other
	Total	¥9680RMB	

Therefore, the reliable hardware modules with long term support from open source communities (Community, 2020; Pixhawk | The hardware standard for open-source autopilots, 2020), as well as the commonly used standard RC racing sailboat form, make our hardware architecture to be attractive in the long term.

### Hardware Architecture

**FIGURE 4:**
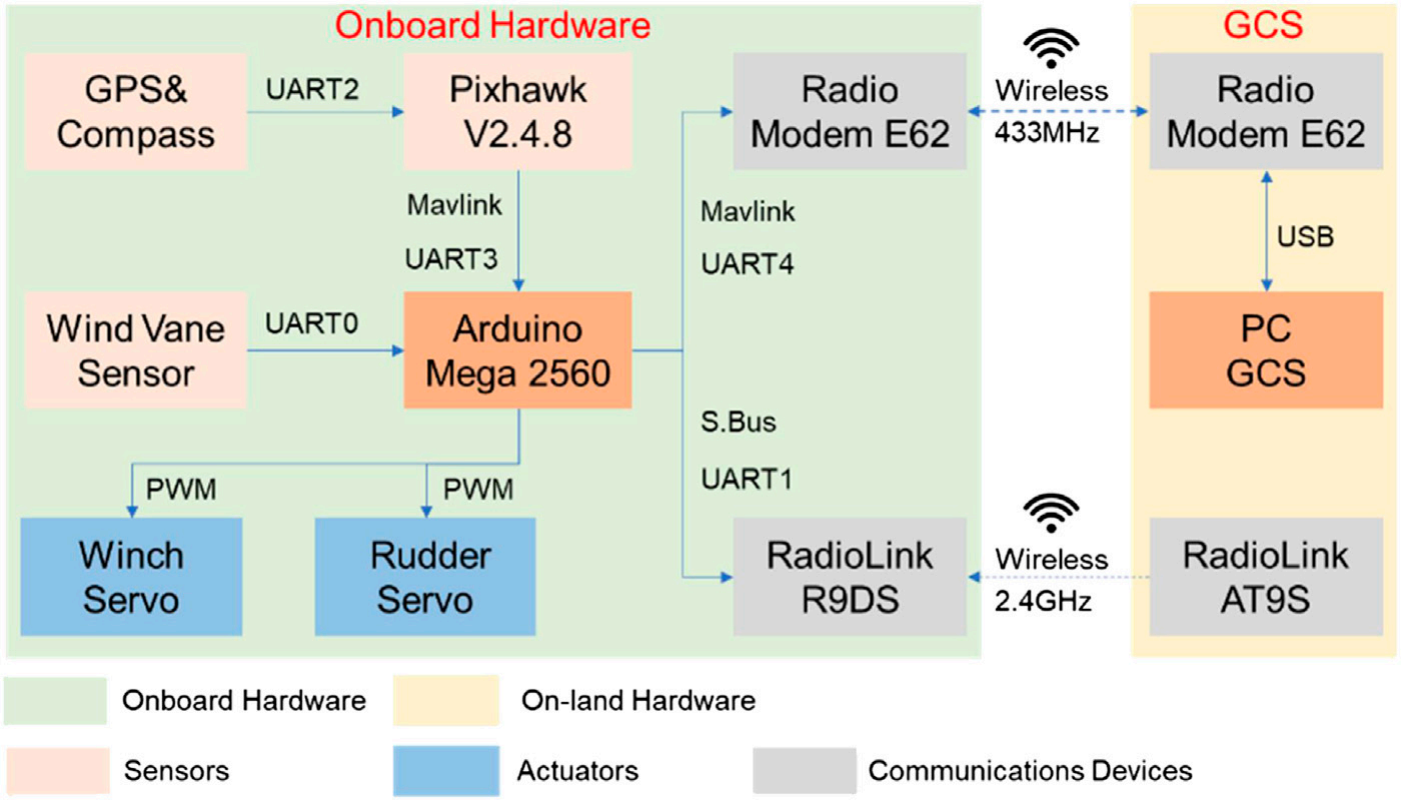
Hardware connection diagram.

The overall hardware connection is shown in Figure 4. It can be divided into two parts: onboard one and on-land one. The onboard hardware modules can be subdivided into sensing unit (in orange color), actuator unit (in blue color), communication unit (in gray color), and control unit (Arduino Mega 2,560). Among these units, an accurate but cheap sensing unit is extremely important for robotic sailboats. Thus, the Pixhawk integrated with GPS and compass sensors is selected as an appropriate scenario to output smooth rigid body six DOF (degree of freedom) states. Extended Kalman Filter technology is used to fuse the GPS, compass, and IMU sensors, so that Arduino users could get reliable states’ feedback from the sensing unit. A moving average filter in Arduino is used to treat the raw measurement from the wind vane sensor. The actuator unit includes the winch servo for sail angle control and the rudder servo for rudder angle control. The communication unit involves the 433 MHz radio modem E62 for data exchange, and 2.4 GHz radio receiver R9DS for remote human operation. Additionally, more payload sensors could be incorporated in our flexible hardware architecture, attributed to the rich peripheral hardware resources (e.g., GPIO, UART, CAN) on Arduino and Pixhawk control board.

## Autonomous Sailboat Software Design

### Modular Structure of Software Firmware

**FIGURE 5:**
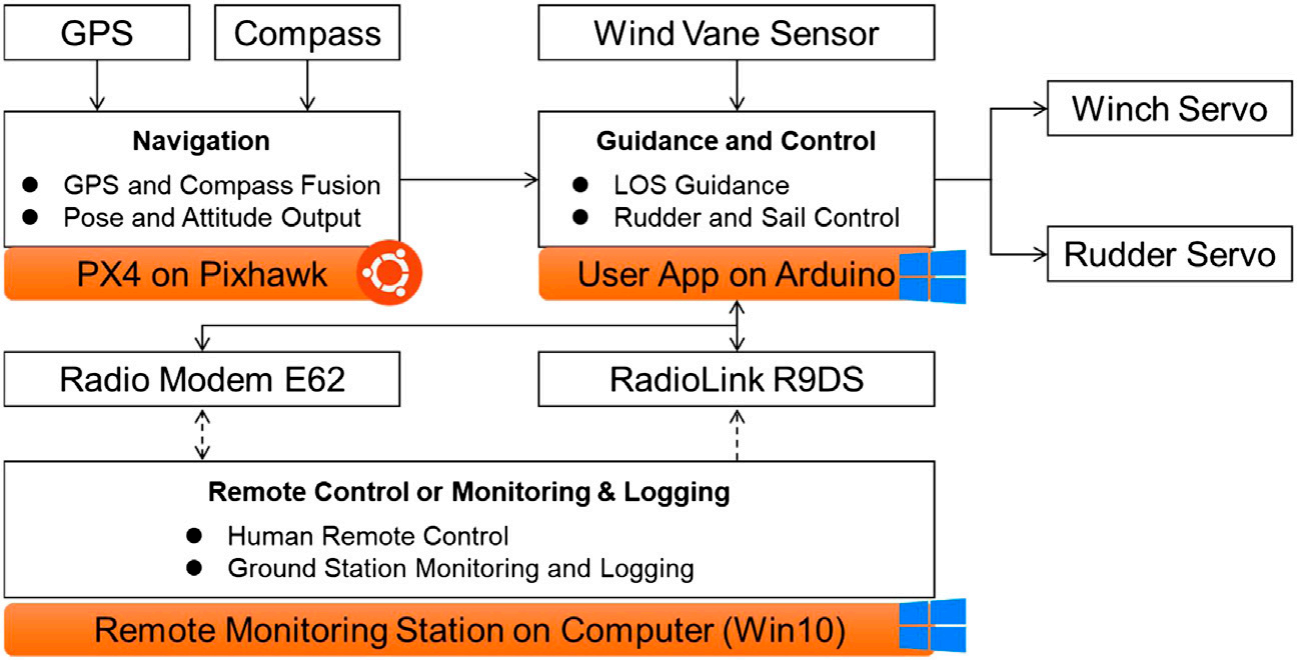
Illustration of functionality of microcontrollers and remote computer.

To govern an autonomous surface vehicle, the basic functionalities can be two parts: path planning and path following (Liu et al., 2016). Generally, a guidance, navigation and control (GNC) architecture is introduced for autonomous marine crafts to realize two parts functionalities (Fossen, 2011). In view of the hardware architecture, the functionalities of each programmable controller and on-land computer are displayed in Figure 5. A ground control station (GCS) was made and running in the on-land computer, so that developers could monitor and keep a log of the autonomous sailboat in real-time. The PX4 firmware running on Pixhawk is an ideal software package to estimate sailboat states. Although the PX4 firmware is commonly used as a flight management unit for drones (Meier et al., 2015), it plays a role as a state observer for the sailboat. In such a case, users have no need of programming in Linux (e.g., Ubuntu 16.04LTS) with complicated real-time operating system (RTOS) code, and could freely get the whole PX4 firmware via the Pixhawk official website. Arduino users only focus on guidance and control algorithms using a simple integrated development environment (IDE) software running on the Windows platform. Therefore, the overall programming difficulties would be acceptable and can be solved without a high threshold of computer programming knowledge.

**FIGURE 6:**
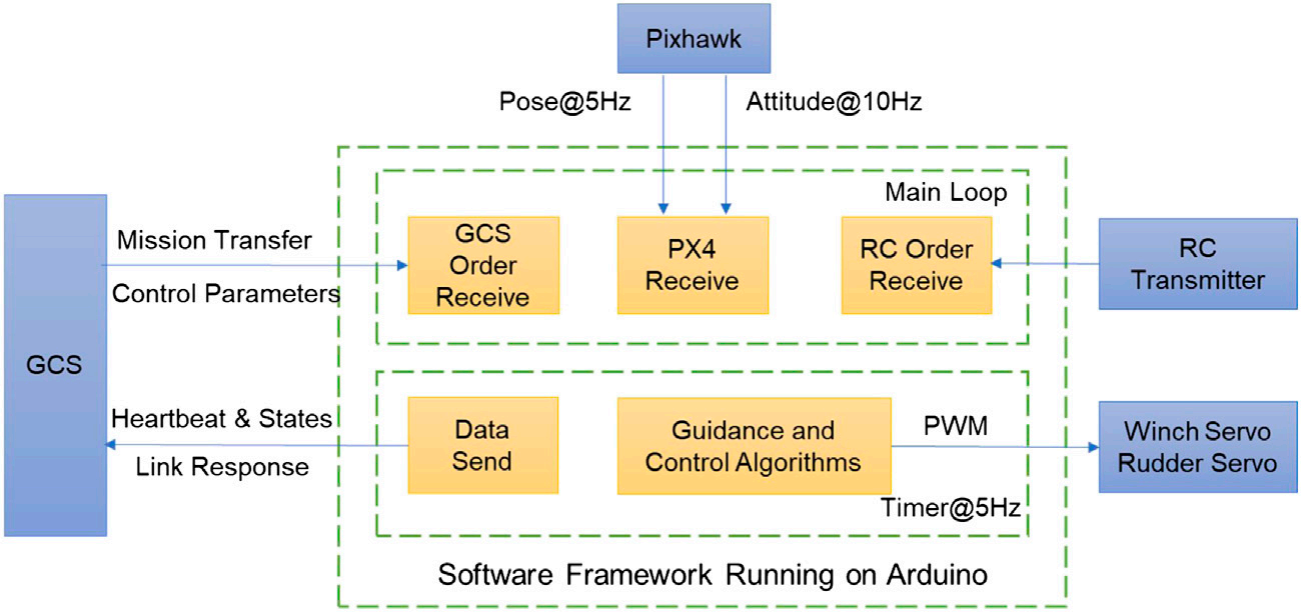
Software framework running on Arduino Mega 2,560 microcontroller.

Due to the easy to use characteristic of Arduino and its libraries, here the Arduino Mega 2,560 microcontroller was designed as the main controller (as shown in Figure 4). The software framework running on this controller is shown in Figure 6. Based on the tasks’ characteristic, two loops, main loop and timer loop, are considered to build the basic embedded software structure. For the main loop, the receive tasks from GCS, Pixhawk and RC transmitter are programmed in a main while (1) function loop. By contrast, the tasks of performing algorithms, as well as data send back to the GCS, need more strict running cycles. Therefore, a timer loop is introduced to conduct the guidance and control algorithms, as well as data send function. The period of the timer loop is mainly dependent on the sailboat inertia characteristics and the time-variant external environment. For example, the pulse width modulation (PWM) commands for actuators were periodically sent in the timer loop at 5 Hz. So, the robotic sailboat could response timely from actuators’ periodic control forces and moments. Meanwhile, the GCS could receive the data related to sensing and control at the same frequency, which would be helpful for further analysis and optimization of the control performance.

### Robust and Lightweight Mavlink Protocol

To ensure the reliable data exchange between the GCS and sailboat, the micro air vehicle link (Mavlink) protocol is tested. Compared with other wireless communication protocols, the mavlink protocol is widely used for unmanned mobile robots (Koubâa et al., 2019). The Mavlink v1.0 frame is shown in Table 4. The total length of one frame can vary from 8 to 263 bytes, which is dependent on the payload messages. The first byte of the Mavlink v1.0 frame is STX with the value of 0xFE, which indicates the start of a new packet. From the second to six bytes, the basic information about the payload was described, including length, object ID, and message ID. Then, the actual payload message is packed based on the message ID. Finally, two bytes checksum (low byte CKA and high byte CKB) is included to ensure the successful message transmission. Due to the lightweight characteristic of the Mavlink v1.0 frame, it can be transferred via several types of hardware interface resources, including UART, 433 MHz radio modem, and even Zigbee modem. Therefore, in our system (as shown in Figure 3), it was used to link the Pixhawk and Arduino, and the Arduino and GCS. Open-source third-party libraries of the mavlink protocol favor ease to use over efficiency for Arduino users.

**TABLE 4:** Mavlink v1.0 Frame (n = 8–263 bytes).

Number	1	2	3	4	5	6	7	…	N-2	N-1	N
Name	STX	LEN	SEQ	SYS	COMP	MSG	PAYLOAD			CKA	CKB

## Autonomous Sailboat Sailing and Controlling

### Points of Sailing

**FIGURE 7:**
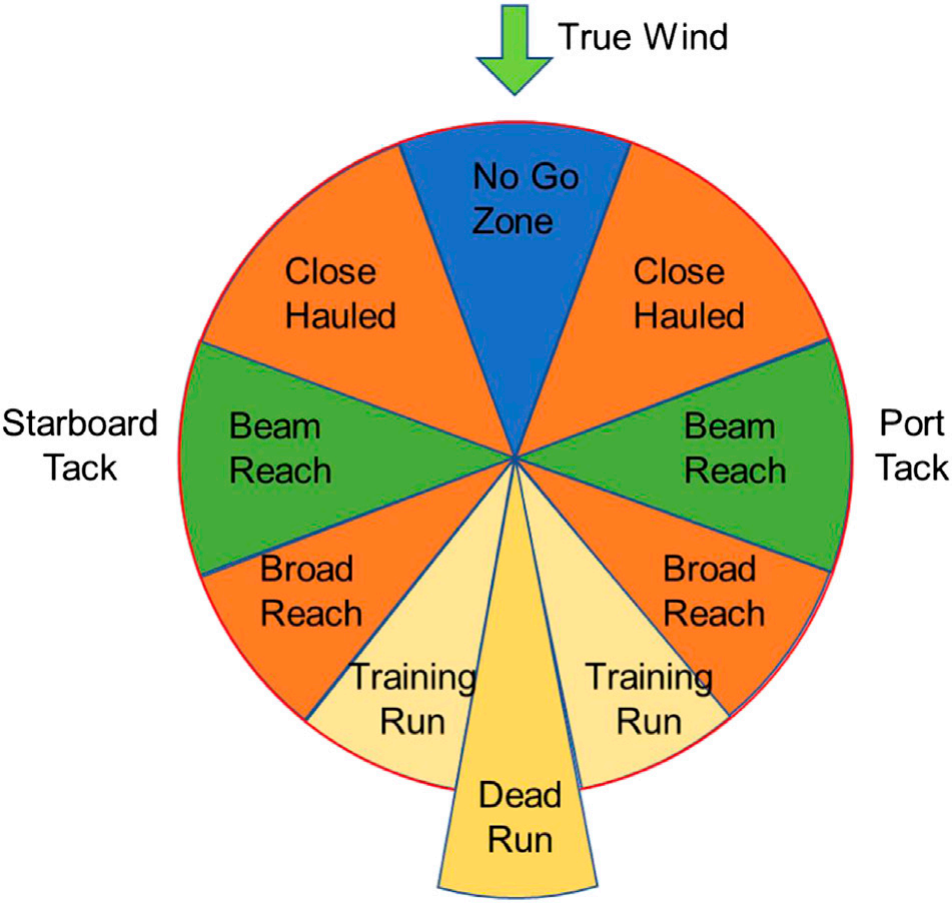
A Point of Sail for guiding autonomous sailing.

Compared with screw-propelled boats, sailboats have a unique sailing characteristic at wind propulsion. As shown in Figure 7, it describes the sailboat course direction change under true wind direction from top to down. Based on the sailboat aerodynamics (Boehm, 2014), the sailboat propulsion power or course direction is closely related to the true wind direction over the surface. For example, about 30° on either side to the true wind is a no-go zone for the sailboat, in which sailboat needs zigzag maneuvering to move forward (Silva Junior et al., 2020). At 180 degrees off the true wind (sailing in the same direction as the true wind) is the dead run, in which the sailboat may also need maneuvering to speed up. Generally, when the sailboat is in the direction ranging from a close-hauled to a training run, the sail would act like a wing, where the sailing force propels the sailboat to move ahead as well as causes significant side force. Therefore, special attention should be paid to navigate the sailboat to the predefined waypoints.

### Simplified Controller Design

**FIGURE 8:**
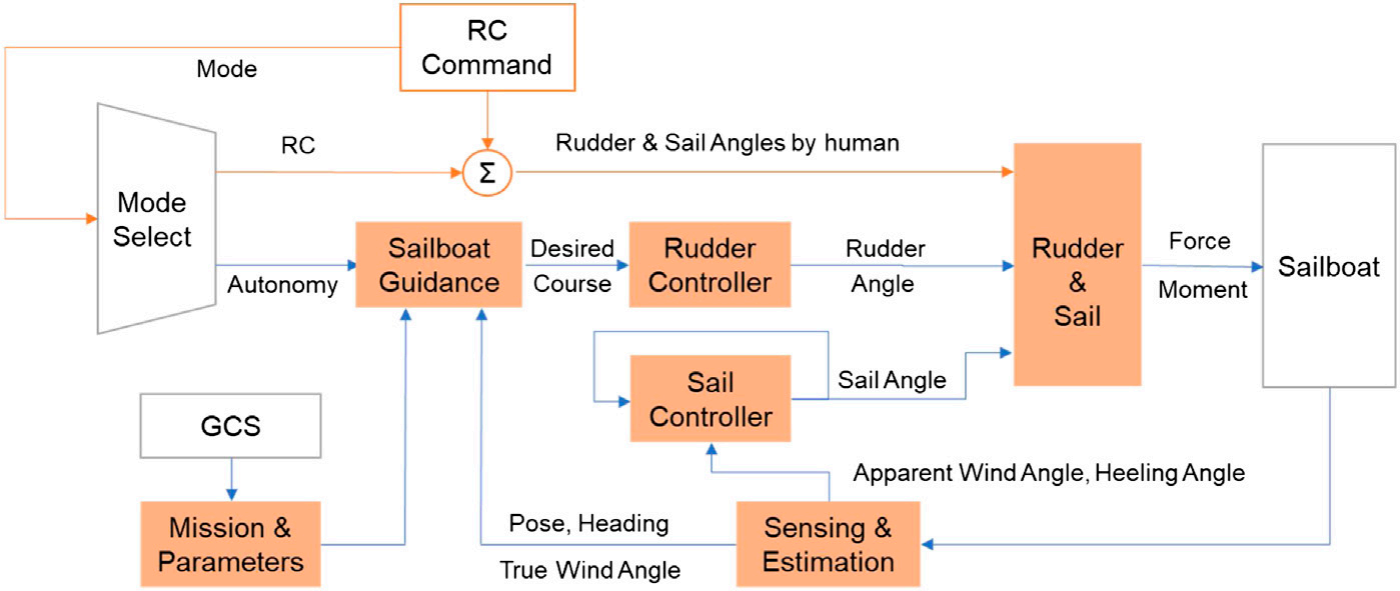
Guidance and control structure of SAILAMRS sailboat.

According to the software framework of the main controller (shown in Figure 6), the guidance and control structure was proposed with the functionality of RC manual control and autonomous sailing. As shown in Figure 8, the modes of RC manual control and autonomous sailing could be switched with the RC command from the radiolink AT9S. For the RC mode, actuators’ orders would be directly connected to the RC commands from human operators, and GCS could be used as an auxiliary monitoring station. For the autonomous mode, human operators used the GCS to plan the mission and parameters, and then waypoints were generated and downloaded to the Arduino Mega 2,560 microcontroller. After that, a sailboat guidance module and two controller modules of rudder and sail were designed to realize the path following functionality. Considering the controller to be easy to use and easy to tune, decoupled controllers were designed for servo actuators of both rudder and sail.

**FIGURE 9:**
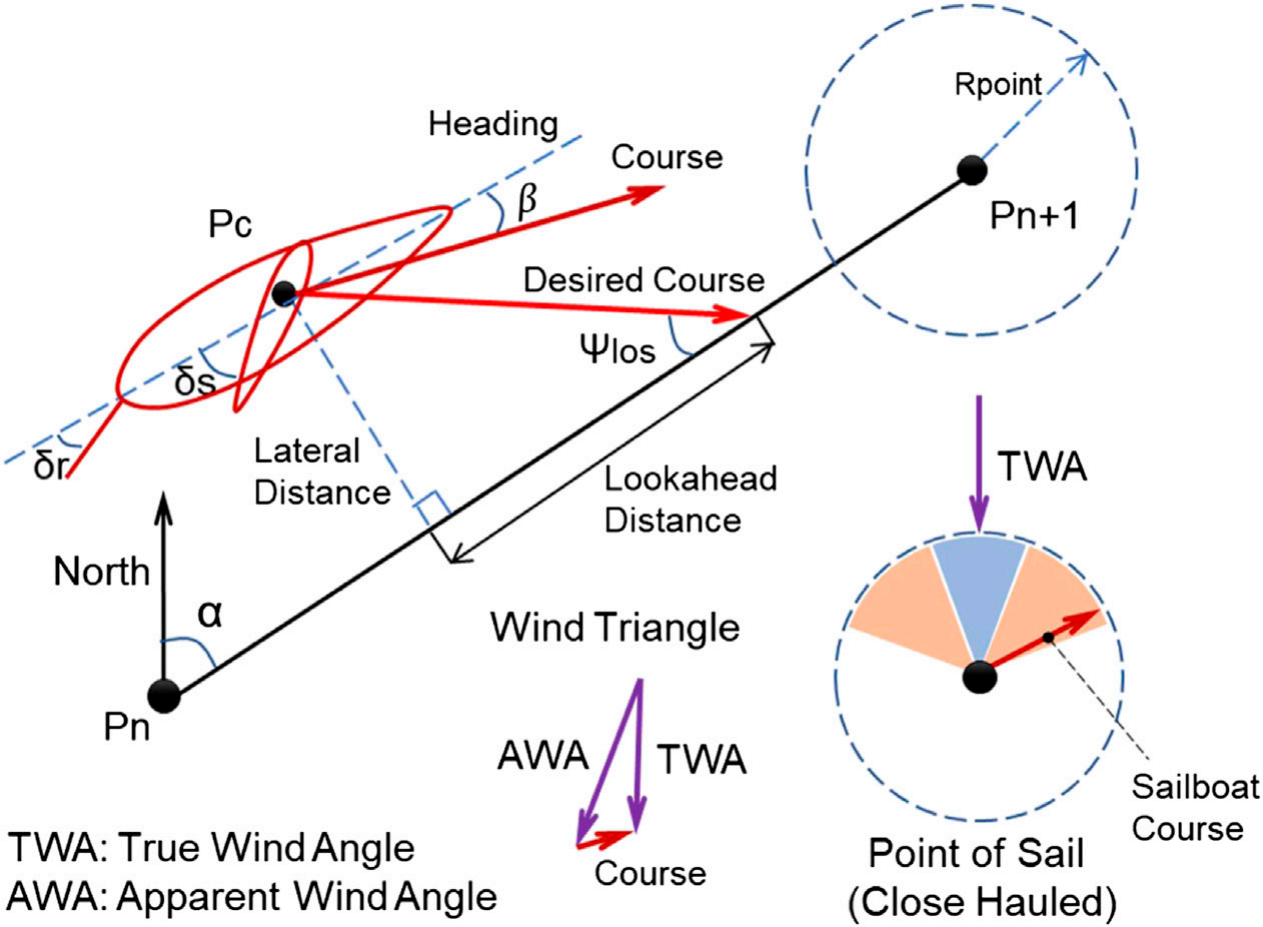
Autonomous sailboat sailing and guidance principle (analysis at horizontal plane).

**FIGURE 10:**
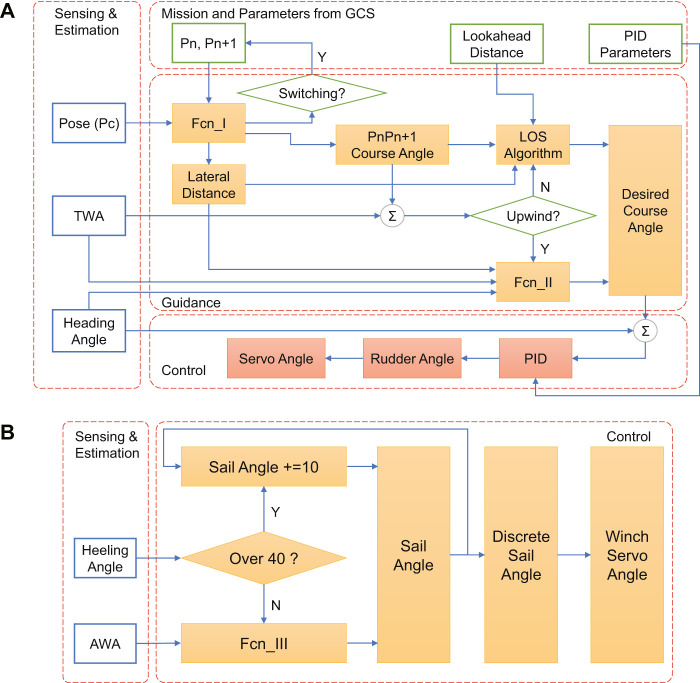
Detailed block diagram of control architecture, **(A)** Sailboat guidance and rudder controller, **(B)** Sail controller.

Similar to other autonomous surface vehicles, a line of sight (LOS) guidance algorithm was used to generate the desired course angle, which was then followed by a PID-based rudder controller to track the planned path. The kinematic principle of the guidance algorithm is illustrated in Figure 9. And the corresponding control block diagram of the rudder is also displayed in Figure 10A. Based on the geometrical relationship of last waypoint (Pn), next waypoint (Pn + 1), and current position of the sailboat (Pc), it is easy to calculate the lateral distance, desired path course angle (α), and the distance to the next waypoint (PcPn+1), which was realized in the Fcn_I block. Then, considering the predetermined lookahead distance parameter, the lookahead angle (Ψlos) could be obtained via Eq. 1.

$$\Psi_{\text{los}}=\arctan\left(\frac{\text{Lateral}\;\text{Distance}}{\text{Lookahead}\;\text{Distance}}\right),$$ (1)

$$\Psi_{\text{error}}=\Psi_{\text{desired}}-\Psi_{\text{measured}}=\Psi_{\text{los}}+\alpha -\beta -\Psi_{\text{measured}}.$$ (2)

So, the heading error (Ψerror) could be calculated via Eq. 2. Generally, the sideslip angle (β) can be neglected due to the slow side motion compared with the surge motion. However, special attention should be paid to the point of sail (shown in Figure 7), and the guidance algorithm for the case of upwind was a bit different. If the sailboat was sailing toward the no-go zone, a zigzag maneuvering action (Erckens et al., 2010) was taken to reach the next waypoint, which was realized in the Fcn_II block. For the Fcn_II function, a predefined desired course angle of 35° relative to the true wind direction was set at each side tack, and the triggering condition to switch from −35° to +35° (relative to true wind direction, not to North direction) was based on the lateral distance of sailboat. So, in the upwind case, the autonomous sailboat could move forward by following the predefined desired course angles, and performing simply but effective tacking strategy. As for the heading following, the PID controller was designed to output rudder angle at heading errors. Additionally, based on the mechanical transmission from the rudder servo to the real rudder angle, the angle servo mapping function could be obtained via real test in Eq. 3. Therefore, the rudder servo (*δ*_servo_) could be commanded by the PID controller output (*δ*_rudder_). After the sailboat reached the circle of the next waypoint with a radius of Rpoint, the sailboat turned to track the next one until the final target point.

$$\delta_{\text{servo},\text{rudder}}=1.1\delta_{\text{rudder}}+93\;\left(-40\leq \delta_{\text{rudder}}\leq 40\right).$$ (3)

Meanwhile, the sail angle was simultaneously controlled based on the apparent wind angle and sailboat heeling angle (shown in Figure 10B). Due to the side force and moment induced by sail, a significant heeling effect would be observed, which was directly related to the sail angle (Boehm, 2014). Thus, a simple linear sail angle (*δ*_sail_) controller was designed via Eq. 3, which was easy to be implemented in the Fcn_III block. Due to the mechanical structure constraint, the upper and lower bounds of the sail angle were set to 5 and 85°. From Eq. 3, it can find that the larger the incident flow AWA, the bigger the desired sail angle. Then, the winch angle servo mapping function could also be built via real test in Eq. 5. Therefore, the final sail angle commands would be implemented by winch servo accurately.

$$\delta_{\text{sail}}=\{\begin{array}{ll} 5 & \left(\left|\text{AWA}\right|<15\right)\\ 0.5\;\text{AWA}-2.5+\left(\left|\text{heeling}\;\text{angle}\right|/2\right) & \left(15\leq \left|\text{AWA}\right|\leq 175\right)\\ 85 & \left(\left|\text{AWA}\right|>175,\delta_{\text{sail}}=85\right) \end{array},$$ (4)

$$\delta_{\text{servo},\text{winch}}=-0.74\delta_{\text{rudder}}+112\;\left(5\leq \delta_{\text{sail}}\leq 85\right).$$ (5)

## World Robotic Sailing Championship Mission-Oriented Verification in the Lake Test

Based on the previous 12 events of WRSC competitions (Robotic Sailing: Home, 2020), the WRSC could be summarized into four challenges, including fleet race, station keeping, area scanning and obstacle avoidance. A pre-defined course area would be set based on the weather conditions at sea. To test the SAILAMRS sailboat’s performance and its hardware and software algorithms, we did several field trials at Yujia Lake in Wuhan, China.

**FIGURE 11:**
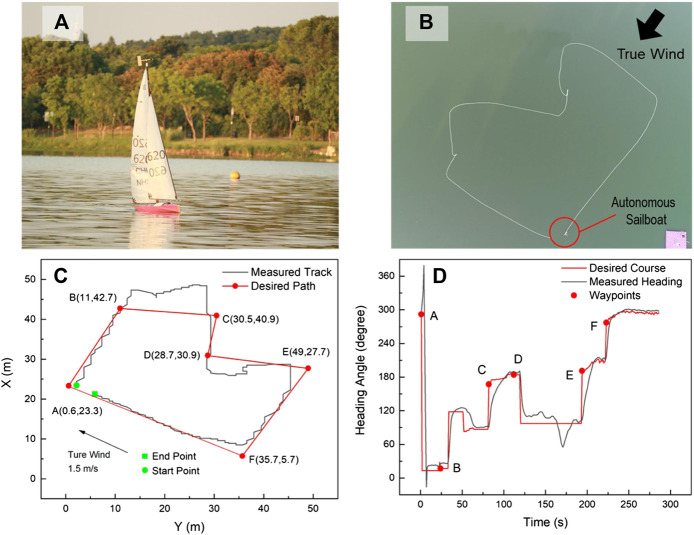
Fleet race capability field tests of SAILAMRS sailboat: **(A)** real photo on lake, **(B)** actual trajectory from camera drone, **(C)** path following performance, **(D)** heading tracking performance.

As shown in Figure 11, the fleet race capability of SAILAMRS sailboat was tested at a true wind speed of 1.5 m/s. A total of six waypoints were planned using the GCS. Figures 11A,B show the real photo of the field test, in which a DJI camera drone was used to capture the actual trajectory. The field test’s data logging was plotted and compared with the planned path in Figure 11C. Based on the knowledge of the point of sail (shown in Figure 7), it can be observed that from waypoint B to waypoint E, a zigzag maneuvering action was selected to push the sailboat toward the upwind direction. However, the path following accuracy during this action would hard to be guaranteed, due to the weak maneuvering capability and relatively low sailing speed in the no-go zone area (Zhang et al., 2018). While, from waypoint A to waypoint B or waypoint E to waypoint F, the case of beam reach was encountered for the sailboat, in which the sail would act like an airplane wing, and contribute efficiently in the heading direction. Then, the last part was from waypoint F to waypoint A. The sailboat would be mainly pushed forward directly by the wind resistance of the main and jib sails. Additionally, as shown in Figure 11D, from the viewpoint of heading control, obvious tracking errors could be observed during the sailboat switched to the next waypoint. After a short adjustment process, the rudder controller could follow the desired course angle well.

**FIGURE 12:**
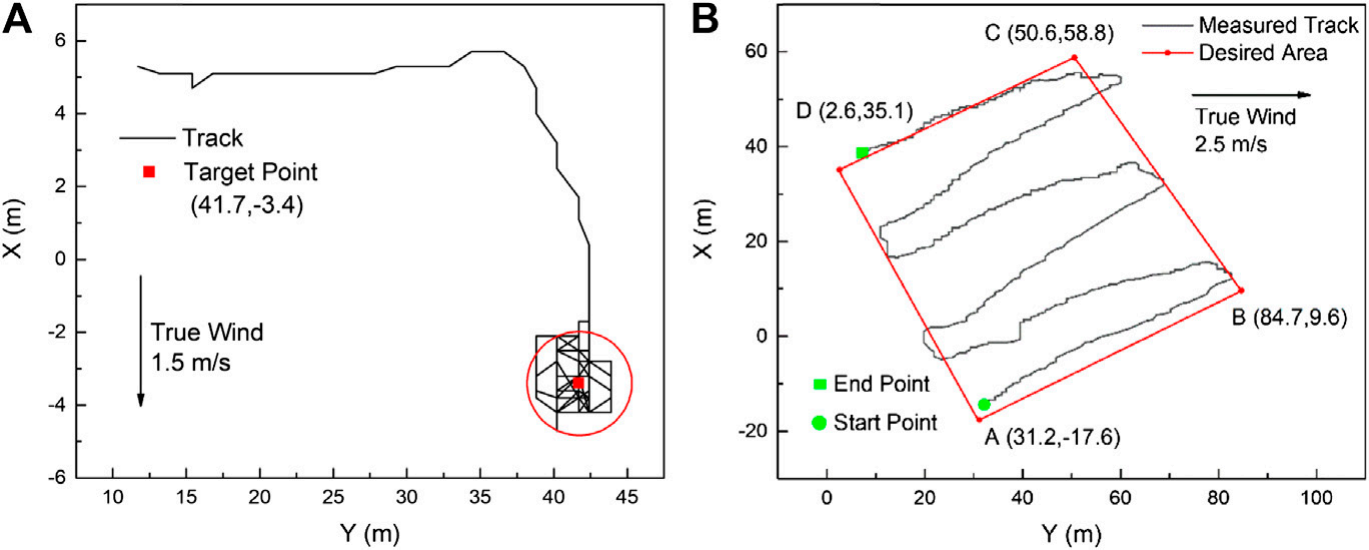
Sailing tracking performance: **(A)** station keeping, **(B)** area coverage.

More field test results are shown in Figure 12. As shown in Figure 12A, the station keeping capability was checked under a true wind speed of 1.5 m/s. When the sailboat reached the target waypoint, it could keep its station in a circle of less than 3.2 m (about 3 times of sailboat length). This good station keeping performance would be helpful to perform some fixed-point ocean observation using the sailboat platform. Furthermore, due to the sailboat’s outstanding endurance, the area coverage test was also verified in Figure 12B. Based on the GPS track line and desired sweeping plan, the autonomous sailboat could follow the mowing path under a true wind speed of 2.5 m/s, although the tracking performance around the turning corner was still needed improvement. Therefore, a sweeping survey using an unmanned sailboat would be expected in the near future.

## Conclusion

Inspiring researchers and students to widely participate in science and technology aspects of robotic sailboat research is a laudable goal, in view of ocean science worldwide data demand. In this paper, a low-cost and easy-access unmanned sailboat platform was designed. A commonly-used 1-m class RC racing sailboat was upgraded to an autonomous one. Open-source and reliable hardware modules, like Arduino Mega 2,560 and Pixhawk V2.4.8, were employed to set up the basic hardware structure. A modular software firmware architecture was designed for Arduino microcontroller, in which users could easy to use and learn with rich libraries. Reliable and mature mavlink protocol was used to provide communication among different module parts. Based on the general point of sail principle, the guidance and control algorithms with LOS path following and upwind tacking were designed and verified in lake tests. Several WRSC competition tasks were checked using the proposed autonomous sailboat with the good path following capability.

Since the autonomous sailboat is a suitable wind-powered ocean observation platform, its research and related unmanned sailing technologies still need further investigation. More effort in terms of sailboat velocity control and optimized sail control would be made in follow-up studies.

## Data Availability

The original contributions presented in the study are included in the article/Supplementary Material, further inquiries can be directed to the corresponding author.
